# Chest CTA in children younger than two years – a retrospective comparison of three contrast injection protocols

**DOI:** 10.1038/s41598-019-54498-z

**Published:** 2019-12-02

**Authors:** Eszter Nagy, Sebastian Tschauner, Robert Marterer, Regina Riedl, Erich Sorantin

**Affiliations:** 1 Division of Paediatric Radiology, Department of Radiology, Medical University Graz, Graz, Austria; 2 Institute of Medical Informatics, Statistics and Documentation, Medical University Graz, Graz, Austria

**Keywords:** Paediatric research, Paediatric research

## Abstract

To obtain the highest diagnostic information with least side effects when performing thoracic CT angiography (CTA) is challenging in young children. The current study aims to compare three contrast agent (CA) injection protocols regarding image quality and CA characteristic: a standard CTA, a fixed-bolus delay protocol, and the “microbolus technique (MBT)” developed in our institution. Seventy chest CTA scans of patients (<2 years) were divided into three groups. MBT was applied in group I, the standard protocol in group II and a fixed bolus delay in group III. Objective image quality was assessed by measuring peak enhancement, image noise, signal-to-noise (SNR) and contrast-to-noise ratios (CNR). Two observers scored subjective image quality and artifacts. Significantly lower amounts of CA (mean ± SD) were used in the MBT group compared to Group II (9.0 ± 3.7 ml vs. 12.9 ± 4.5 ml). A lower, but still diagnostic (>400 HU) enhancement was registered in all major thoracic vessels in group I without significant differences regarding SNR and CNR in most regions (p < 0.05). The best scores for image quality and artifacts were reached in group I. All three chest CTA contrast injection protocols offered diagnostic vessel enhancement in young patients. MBT was associated with reduced image artifacts and less injected CA.

## Introduction

Contrast-enhanced CT imaging has a particularly important role in the evaluation of thoracic and vascular malformations as well as lung and airways disorders in young children^1^. The application of iodinated contrast agent has a potential risk on kidney function, although contrast-induced acute kidney injury is recently deemed to be overstated in the literature and overestimated by clinicians in the adult patient population^2^. The literature regarding contrast-induced nephrotoxicity in pediatric patients is limited, especially in neonates^3^. Nevertheless, a recently published study supports the presence of this condition with an incidence of 10.3%^4^. Besides kidney function impairment, CA osmotic load may have a more relevant impact on fluid homeostasis in neonates and infants because the osmolarity of the widespread used iodinated CAs with an iodine concentration of 300–320 mgI/ml^5^ still exceed the osmolarity of the blood serum.

To get the highest possible diagnostic information in one examination, radiologists need to consider the anatomical and physiological differences between adults and small children^6^. The fast circulation system requires an adapted contrast agent application protocol to catch the vessel enhancement properly. The less calcified skeletal system and less muscle mass make high-energy tube voltage settings unfavorable. Lower peak kilovoltage (e.g., 80kVp)^7–11^ not only reduces the radiation dose but improves tissue contrast, thus facilitating a reduction in CA volume^12^. An adapted contrast application protocol enables a simultaneous enhancement in both arterial and venous parts in different vascular and cardiac malformations^13^.

Due to the heterogeneous recommendations and lack of large comparative studies^14^, three different chest CT CA injection protocols were in use within the last decade since 320-slice volume CT was introduced in our institution (tertiary pediatric center). The primary protocol in the first years of the study period used a common bolus tracking technique with undiluted CA followed by saline flush^15–17^, frequently resulting in unsatisfying image quality due to excessive vessel enhancement. The introduction of a new dual-flow power injector^18^ facilitated the modification of this protocol into a continuous and simultaneous application of CA as well as physiological saline (PS) until the threshold was reached. This approach enabled imaging the arteries and veins in one scan and was named “microbolus technique” (MBT). As a third contrast protocol, fixed bolus delay was applied for specific clinical indications.

The purpose of the study was to perform a retrospective assessment of  these three CA application protocols with regard to injected CA volume, contrast enhancement in several representative thoracic regions, overall image quality and CA related image artifacts.

## Methods

The study protocol was approved by the Ethics Commission of the Medical University of Graz (approval number EK 29-390ex16/17), and all study-related procedures were performed in accordance with relevant guidelines and regulations. Due to the retrospective character of the study, the local review board required no written informed consent from the parents/guardians of study participants. The authors declare no conflicts of interest.

### Patient population

**Figure 1:**
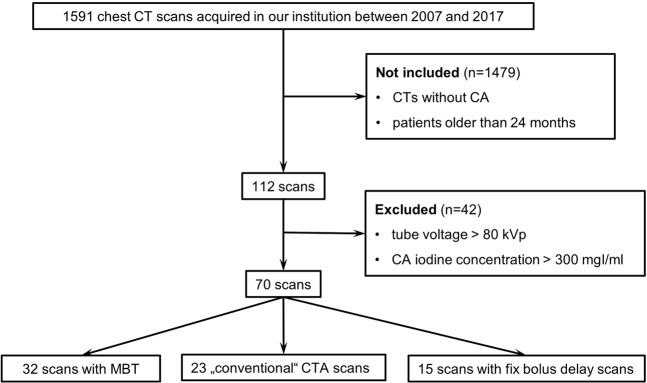
Study population flow chart.

The patient population is depicted in Fig. 1. 70 scans from 58 unique sequential patients younger than two years, who underwent chest CTA between 2007 and 2017, with a tube voltage setting of 80kVp and CA Iodine concentration of 300 mg/ml were included. In 9 patients, a follow-up imaging was needed depending on the clinical decision. Since cardiovascular status at the specific moment of an examination can show large individual deviations, we handled repeated examinations of these 9 patients independently from each other in the statistical analyses. According to the CA application protocol, scans were divided into three groups as listed below. For all scans, patient age and weight were available. According to ACR–ASER–SCBT-MR–SPR practice parameter for the performance of pediatric computed tomography^1^ underlying diseases of patients were categorized into six groups: diseases of the extracardiac vessels, of the heart, of the tracheobronchial systems, of the lung, of the mediastinum and miscellaneous. Patient characteristics are summarized in Table 1. The effective diameter was calculated manually according to AAPM 204 (American Association of Physicists in Medicine)^19^.

**Table 1:** Patient characteristics.

	Group I (n = 32) (mean ± SD)	Group II (n = 23) (mean ± SD)	Group III (n = 15) (mean ± SD)	p-value
**Patient age (months)**	5.8 ± 5.2	7.7 ± 6.4	7.4 ± 5.1	0.411
**Patient weight (kg)**	5.7 ± 2.5	6.8 ± 2.6	7.0 ± 2.0	0.149
**Effective diameter (mm)**	126.4 ± 14.0	119.8 ± 16.5	129.1 ± 14.0	0.108
**Underlying diseases**				
**Extracardiac vascular**	**10 (31.2%)**	**10 (43.5%)**	**1 (6.7%)**	—
Vascular rings	1	1	—	—
Pulmonary vein abnormalities	3	6	—	—
Coarctation of aorta	2	1	1	—
Sequester	3	2	—	—
Miscellaneous	1	—	—	—
**Cardiac**	**5 (15.5%)**	**4 (17.4%)**	—	—
Tetralogy of Fallot	2	1	—	—
Univentricular heart	1	—	—	—
Miscellaneous	2	3	—	—
**Tracheobronchial**	**2 (6.3%)**	**3 (13%)**	**1 (6.7%)**	—
**Mediastinal**	**2 (6.3%)**	—	—	—
**Lung**	**11 (34.4%)**	**4 (17.4%)**	**11 (73.3%)**	—
Congenital	8	4	2	—
Infection	3	—	3	—
Malignancy	0	—	6	—
**Miscellaneous**	**2 (6.3%)**	**2 (8.7%)**	**2 (13.3%)**	—

### CT examination

CT examinations were performed on an Aquilion One 320 row scanner (Toshiba Medical Systems Corporation, Otawara-shi, Japan). Automatic exposure control (AEC) was consistently activated, while electrocardiographic gating was disabled. Helical and volume scan modes were both applied^20^. Adaptive iterative reconstruction (AIDR3D^®^) has been available since a system update in 2012. In the majority of the cases, axial images were reconstructed in 2 and 3 mm slice thicknesses in soft tissue and lung kernels at an increment of 50% of slice thickness, depending on the clinical question and the decision of the responsible radiologist. Reconstruction kernels were recommended by the manufacturer according to the technical setup of the machine. All technical parameters are summarized in Table 2. All examinations were performed in sedation.

**Table 2:** Technical data of the included CT scans.

	Group I (n = 32)	Group II (n = 23)	Group III (n = 15)	p I - II	p I - III	p II - III
**Tube current** **(mAs, median, 95% CI)**	50.0 (52.1, 74.0)	105 (81.5, 32.3)	90.0 (69.7, 113.2)	0.002	0.046	1.000
**Exposure time** **(ms, median, 95% CI)**	500.0	500.0 (486.0, 504.9)	500 (461.7, 662.7)	1.000	0.021	0.006
**Effective tube current** **(mAs, median, 95% CI)**	25.0 (26.9, 40.2)	58.0 (44.1, 67.9)	45.0 (−171.2, 636.1)	0.001	0.036	1.000
**Scan length** **(cm, median, 95% CI)**	13.8 (−12.2, 88.3)	13.7 (11.9, 14.5)	12.2 (11.3, 15.1)	—		
**AIDR active (n)**	24 (75%)	6 (26%)	3 (20%)	—		
**Volume CT (**n)	28 (87%)	21 (91%)	9 (60%)	—		
**DLP (mGy.cm, mean ± SD)**	41.2 ± 26.1	54.1 ± 34.1	40.9 ± 29.8	—		
AIDR active	35.3 (27.2, 50.7)	38.8 ± 34.9	5.7 (−40.4, 79.8)	1.000	0.530	0.621
No AIDR	47.9 ± 20.2	59.5 ± 33.1	46.3 ± 29.5	0.638	0.991	0.470
**CTDI**_**vol**_ **(mGy, mean ± SD)**	2.1 ± 1.1	3.2 ± 1.6	2.9 ± 1.9	—		
AIDR active	1.8 ± 0.9	1.8 ± 1.4	0.6 (−0.7, 2.5)	0.997	0.274	0.400
No AIDR	3.1 ± 1.3	3.6 ± 1.5	3.5 ± 1.8	0.643	0.820	0.953
**SSDE (mGy, mean ± SD)**	2.7 ± 1.2	4.1 ± 1.9	4.2 ± 2.7	—		
AIDR active	2.4 ± 0.8	2.6 (0.2, 6.3)	1.4 (0.6, 2.8)	0.341	0.564	0.183
No AIDR	3.6 ± 1.6	4.3 ± 1.6	4.9 ± 2.5	0.661	0.313	0.706
**Soft tissue kernel (n)**						
FC17	6 (18.8%)	15 (65.2%)	10 (66.6%)	—		
FC18	26 (81.2%)	6 (26.1%)	3 (20%)	—		
FC14	0	2 (8.7%)	1 (6.7%)	—		
other	0	0	1 (6.7%)	—		
**Slice thickness in soft tissue reconstruction, axial (n)**						
3 mm	29 (91%)	16 (70%)	9 (60%)	—		
2 mm	3 (9%)	5 (22%)	2 (13.3%)	—		
other	0	2 (8%)	4 (26.7%)	—		

### Protocols for iv CA application

**Figure 2:**
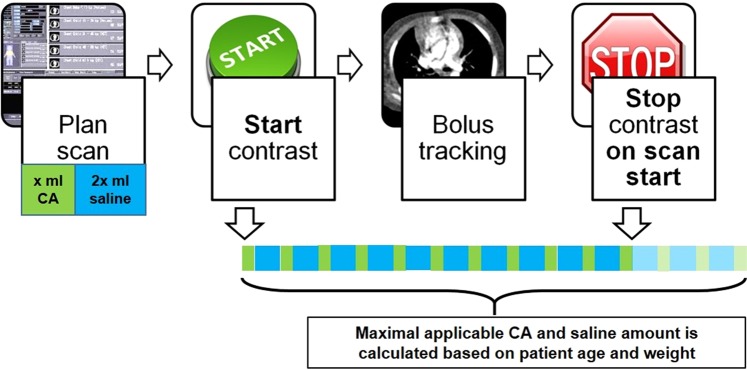
Schematic illustration of MBT. The examination is planned by computing contrast agent amount using a semiautomatic algorithm. A power injector is used to infuse a 2:1 mix of PS and CA. A ROI is placed within the descending thoracic artery; bolus tracking threshold is set 400 HU and the power injector is started. Trigger value of bolus tracking is reached before consuming the whole amount of CA and PS, and thus, the power injector is immediately stopped by the operator and the rest of CA discharged.

In Group I (n = 32), MBT (Fig. 2) was applied to optimize the opacification of major arterial and venous thoracic vessels as well as minimize artifacts. The amounts of CA and PS were prior calculated according to patient age and weight. Both of them were applicated as an alternating bolus with a 400 Hounsfield Units (HU) threshold for bolus tracking. As the threshold delay was reached, the power injector was stopped manually. In this way, the venous structures are supposed to be filled properly, which allows the imaging of arteries and veins in one setting. For bolus tracking, the region of interest (ROI) was placed within the descending aorta in a way to avoid unnecessary gantry movement before the scan^20^. In Group II (n = 23), scans were done using non-diluted CA and PS flush at the end of the CA application. The bolus tracking ROI was placed in the same way as in Group I. In Group III (n = 15) CA was administrated with a fixed bolus delay based on patient age and weight according to an already published strategy^20^. For CA administration, the same power injector (Ulrich GmbH&Co.KG, Ulm, Germany) was used in all groups, which enabled injecting a mixture of PS and CA. The amounts of injected CA (in ml) were recorded.

### Assessment of contrast enhancement

Quantitative contrast enhancement measurements were performed on  a radiological workstation (syngo.plaza VB20A; Siemens Healthcare, Erlangen, Germany) by a radiologist in training with three years of experience (E.N.).

Measurement ROIs were placed in the ascending (AA) and descending (AD) thoracic aorta, aortic arch (Aar), pulmonary trunk (PT), right atrium (RA), right ventricle (RV), left atrium (LA), left ventricle (LV), right upper pulmonary vein (RUPV), right lower pulmonary vein (RLPV), left upper pulmonary vein (LUPV), left lower pulmonary vein (LLPV), superior vena cava (SCV), liver (Li) and para-spinal as well as pectoral muscles (Mu). Measurements in pulmonary veins were performed close to their origin. Mean and standard deviation of CT attenuation coefficients in HU was obtained from every ROI three times and averaged afterward. This represented the characteristic value as well as image noise for that particular region. For each region, the signal-to-noise ratio (SNR) was computed by dividing mean peak enhancement with the standard deviation of the mean peak enhancement of a given region^21^. Contrast-to-noise ratio (CNR) for a particular region was calculated with the following formula: [(mean peak enhancement of that region - mean peak muscle enhancement) / image noise measured in muscle regions]^22^. The homogeneity of contrast-enhancement in one group was tested by comparing the mean density values of all vascular regions.

**Figure 3:**
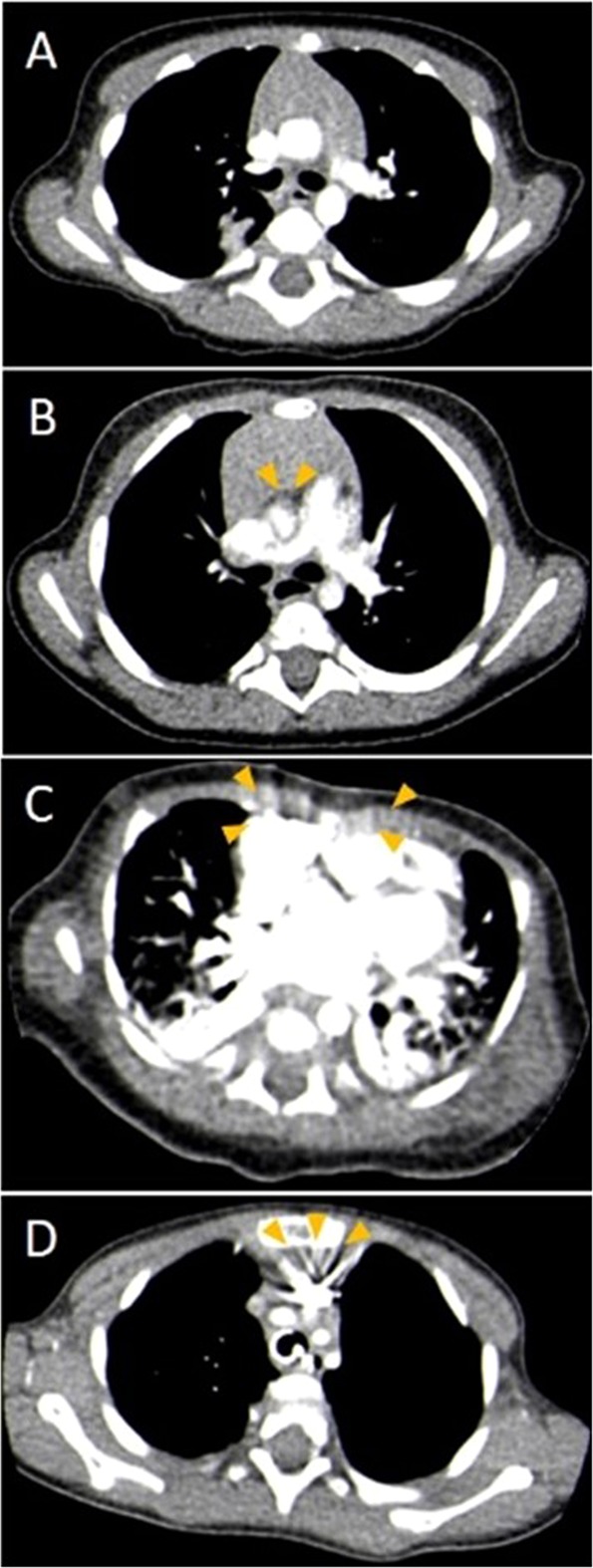
Representative axial CTA images in standard mediastinal window settings (width 400 HU, center 40 HU) to demonstrate different image artefact categories in subjective image analysis (**A**) no artefacts, (**B**) minor artefacts, (**C**) major artefacts, (**D**) extensive image blurring.

Image quality and CA related image artifacts were visually graded by two of the authors (E.N., S.T.) in consensus. A five-grade Likert scale was used for general image quality: score 1, excellent; score 2, good; score 3, acceptable; score 4, poor; and score 5, bad. To complete the assessment, we separately applied a four-grade Likert scale system exclusively for the analysis of CA-related image artifacts: score 1, no artifacts; score 2, minor artifacts; score 3, major artifacts; score 4, marked image noise and extensive image blurring (Fig. 3).

### Statistical analysis

Data are presented as a mean ± standard deviation for continuous variables as well as median and 95% interquartile range for non-continuous variables. For group comparisons of contrast enhancement, linear models were used with adjustment to patient age and weight. To assess the variability of mean vascular enhancement within each group, repeated measurement ANOVA was applied. Fischer’s exact test was used to compare subjective image qualities. P-values lower than 0.05 were assumed to be statistically significant. Statistical analysis was performed with SAS® software version 9.4 (SAS Institute, Cary, NC, USA) and SPSS Statistics version 21 (IBM, New York, United States of America).

## Results

Baseline patient characteristics did not differ significantly from each other (Table 1). CT indications in group I were heterogeneous representing all kind of underlying diseases (Table 1). The main CT indications in group II were extra cardiac vascular and cardiac disorders, while in group III the lung disorders. Examinations to rule out suspected disorders as well as to investigate patients with lymphatic anomalies were included in “miscellaneous”.

Technical data are summarized in Table 2. According to the study protocol, the tube voltage was set at 80 kV. Significantly lower tube current and effective tube current was applied in group I compared to group II and III (for tube current p = 0.002 and p = 0.046 as well as for effective tube current p = 0.001 and p = 0.036, respectively), while no difference was found between group II and III. Volume mode was performed in 87% of group I, in 91% of group II and 60% of group III. Iterative reconstruction was active in 75% of the CT scans in group I, in 26% of group II and 20% of group III. FC17 and FC18 soft tissue reconstruction kernels were applied in most of the cases of the examination, while a different type of kernel was applied in 8.7% of group II and 13.4% of group III. Soft tissue images for quantitative analysis were reconstructed generally in 3 mm axial slices (91% in group I, 70% in group II and 60% in group III).

Dose parameters were analyzed depending on the activation of iterative reconstruction. No significant difference was found in dose length product(DLP), CT dose index(CTDI) and size-specific dose estimation(SSDE) in all groups, independently from the activation of iterative reconstruction.

CA volume is summarized in Table 3. The absolute amount of age- and weight-adjusted CA (mean ± SD) in group I was lower compared to group II and III (9.0 ± 3.7 ml vs. 12.9 ± 4.5 vs. 12.1 ± 5.2 ml), the difference was statistically significant between group I and II (p = 0.005). The weight-adjusted CA dose revealed the same finding as absolute CA volume with significant difference between group I and group II (1.6 ± 0.7 ml/kg and 2.0 ± 0.5 ml/kg respectively, p = 0.012), while no significant difference was seen between group I and group III (1.8 ± 0.7 ml/kg and 1.7 ± 0.5 ml/kg respectively, p = 0.757).

**Table 3:** Baseline data regarding CA.

	Group I (n = 32) (mean ± SD)	Group II (n = 23) (mean ± SD)	Group III (n = 15) (mean ± SD)	p (I vs II)	p (I vs III)	p (II vs III)
CA (ml)	9.0 ± 3.7	12.9 ± 4.5	12.1 ± 5.2	0.005*	0.151	0.316
CA (ml/kg)	1.8 ± 0.7	2.0 ± 0.5	1.7 ± 0.5	0.012*	0.757	0.068
Iomeprol (Iomeron) 521 ± 24 osmol/kg (n)	32 (100%)	8 (34.8%)	4 (26.7%)	—	—	—
Iopamidol (Jopamiro) 616 mosm/kg H2O (n)	0	15 (65.2%)	11 (73.3%)	—	—	—

*Significant difference between groups.

**Figure 4:**
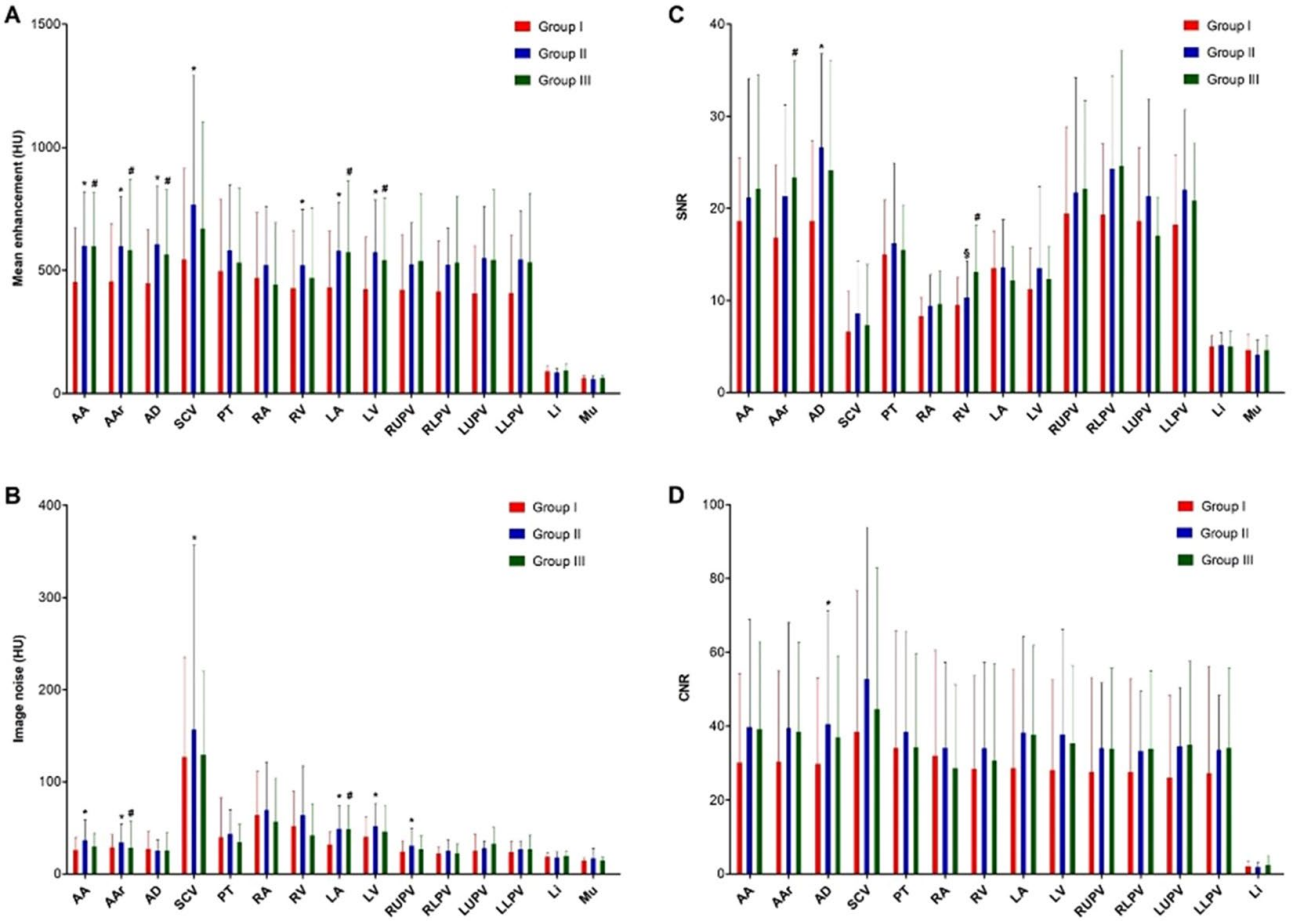
Bar charts of mean enhancement in HU (**A**), image noise in HU (**B**), SNR (**C**) and CNR (**D**) measured in different region. *Significant difference between group I and II, # significant difference between group I and III, § significant difference between group II and III.

The results of the objective image quality assessment are summarized in Supplementary Table S1 and depicted in Fig. 4. In line with the lower amounts of applied CA in group I, a significantly lower, but still diagnostic enhancement (more than 400 HU) was measured in all regions of the thoracic aorta, in left-sided heart chambers, in the right ventricle and the superior vena cava in group I compared to the other groups. Significantly lower amounts of image noise were recorded in group I in the ascending aorta, left-sided heart chambers and in the right upper pulmonary vein compared to group II (p < 0.050). Comparing SNR values of each region, we found significant differences only in the descending thoracic aorta between group I and II (p = 0.005) as well as in the aortic arch and right ventricle between group I and III (p = 0.040 and p = 0.010 respectively). CNR measured over the descending thoracic aorta differed only between group I and II (p = 0.040). The rest of the CNR measurements did not differ between the groups. Groups did not differ concerning contrast enhancement homogeneity in the individual vascular regions (in group I p = 0.076, in group II p = 0.106, in group III p = 0.091).

All of the examinations were diagnostic after adjusting the window settings. Even in case  overall image quality assessment yielded grades 4 or 5, the clinical question could have been answered and no measurement was needed to be repeated. At the assessment of general image quality **(**Table 4**)**, significantly higher scores were found in group I over group II and III (p = 0.020). Evaluation of CA- related artifacts revealed the same findings (p = 0.006). None or only minor artifacts were caused by CA in 78% of the examinations with MBT, while this ratio was 52% in group II and 47% in group III. Noteworthy, that in group I, only 3% were graded with image quality score 4, while image quality score 4 or 5 resulted in 18% of the cases in group II and 33% of the cases in group III.

**Table 4:** Image Quality and CA related artifacts.

General image quality	Group I (n = 32)	Group II (n = 23)	Group III (n = 15)
Score 1	16 (50%)	4 (17%)	3 (20%)
Score 2	10 (31%)	8 (35%)	4 (27%)
Score 3	5 (15%)	7 (30%)	3 (20%)
Score 4	1 (3%)	2 (9%)	5 (33%)
Score 5	—	2 (9%)	—
** CA-related image artifacts**			
Score 1	15 (47%)	2 (9%)	1 (7%)
Score 2	10 (31%)	10 (43%)	6 (40%)
Score 3	6 (19%)	5 (22%)	5 (33%)
Score 4	1 (3%)	6 (26%)	3 (20%)

In the upper part of the table results of general image quality analysis considering the patient motion, and implants. Score 1 is consistent with the best image quality, while score 5 means extensive image artifacts. In the lower part of the table results of image artifact analysis caused by CA alone, where score 1 corresponds to no artifacts with sharp lines while score 4 means extensive streak artifacts.

## Discussion

This paper compared three different CTA contrast agent injection protocols in children younger than two years. We found adequate enhancement of the major thoracic vessels in all three of them. The locally developed MBT using diluted CA showed advantages over undiluted CA and fixed-bolus injection protocols regarding CA amounts and artifact characteristics in patients with the most diverse underlying diseases. Actively stopping the CA application in MBT is a simple additional task for the operator, but it resulted in higher image quality scores.

CTA in neonates and infants remains one of the most challenging tasks in pediatric radiology due to its difficulties in calculation of the scan start delay for CA application. This is particularly challenging in case of cardiovascular malformation, where the simultaneous enhancement in the arteries and veins is required. Besides reducing the radiation burden, the possible side effects of CA application should be avoided, while an appropriate image quality needs to be achieved. Our results indicate a significantly lower CA volume in MBT compared to the routinely used chest CTAs with conventional CTA protocol as well as with fixed bolus delay. MBT allowed a diagnostic enhancement in all of the major thoracic vessels, as well as the highest rate of good image quality, emphasizing the reliability of this method. Despite the low amount of injected CA, SNR and CNR were comparable to the two other CA application protocols. Although the lower effective tube current was used in group I, the lower image noise was measured, which might be explained by the highest rate of the applied standard 3 mm slice thickness, since the reconstruction with thicker slices decreases the image noise^23^. Moreover, the slightly different reconstruction kernel may contribute as well. The registered differences in SNR and CNR in some regions seemed to be occasional in this small patient population.

Subjective image quality was assessed in two ways. First, we considered all image artifacts, even those, which are independent of CA. Second, we rated exclusively the contrast-related image artifacts on a four-grade Likert scale, which supposed to simplify the decision process. In the subjective analysis of CA-relatedartifacts, ten examinations were scored poor, although after modifying image window examinations were still diagnostic, and the clinical question could be answered. The same was true for the two poor-scored examinations in the general image quality analysis. Therefore no examination needed to be repeated.

One possible explanation for better image quality with MBT is attributed to the relatively prolonged application of CA and saline. This does not only lead to the reduction of artefacts, but also to an appropriate enhancement in all major thoracic vessels. On the other hand, the reduced viscosity and osmolarity of CA bolus may contribute to this effect. To prove these assumptions, further studies with animal models should be conducted.

Since the determining factors on CA application are patient age and weight, all results were adjusted for these factors to get a reliable comparison of vascular enhancement^12^. The known physical link between CT density and peak kilovoltage was taken into consideration. Therefore only examinations at 80kVp were included^24,25^. Since it was a CT study, we provided all technical scan data, including the routinely used radiation dose data. However the system updates during study period with the introduction of statistical iterative reconstruction as well as the limited number of patients made dose comparison between the groups impossible.

As mentioned previously, the recommendations regarding CA application protocols in this patient population are heterogeneous, but 2 ml/kg body weight CA volume is commonly advised^15–17,26–34^. Literature data also suggest several alternatives regarding contrast injection protocols. In most of the studies, dual-phase protocol is applied to inject contrast at a constant rate followed by a saline flush, as in group II of our study cohort^16,17,35^. Moreover, to reach an appropriate enhancement in all of the major thoracic vessels, a triphasic protocol can be applied, in which undiluted contrast agent is followed by the admission of 50–60% diluted CA and by a 5–10% diluted CA afterwards^13^. Split bolus techniques to achieve an appropriate enhancement of parenchymal and vascular structures can be cosidered as well^26^, whereas fixed CA delays are not commonly used for angiographies. Dilution of CA was already advised by some authors to reduce streak artifacts particularly in case of an antecubital injection site^13,30,32^. To the best of our knowledge, comparative studies focusing on image quality differences in different contrast agent application protocols are missing.

Besides the favorable effects on image quality, the lower amount of CA could be beneficial in the prevention of potential renal and non-renal side-effects. Although the injected CA with 300 mgI/ml is considered to be safe for the pediatric population, still it exhibits a higher osmolarity (521 ± 24 mOsm/kg) than blood serum, possibly inducing renal tubular damage or potential life-threatening fluid imbalances. Regardless of the lack of evidence for kidney damage after intravenous CA application in newborns and infants^36^, we believe every reduction in CA is beneficial for small patients. An alternative option to reduce side effects is the usage of low-concentrated iodinated CA (e.g. 270 mgI/ml)^21,33,34^. However, this concentration is still considerably higher than those used in MBT (CA dilution to 100 mg Iodine/ml).

In our practice, with the utilization of  a dedicated power injector, no other manipulation has to be done to mix CA and PS, therefore the infection risk is minimized. As an additional feature, the vessel contrast enhancement is prolonged^15^. An earlier study has shown, that the attenuation in heart chambers and the great vessel is independent of injection sites^37^.

Several limitations of our study need to be mentioned. First, the small study population is burdened by the heterogeneity of underlying diseases and the high variability of clinical cases. Due to the retrospective study design, it was impossible to control the presence and type of congenital heart disease, which may cause different hemodynamic status and, therefore, different attenuation of the heart chamber and great vessels. Secondly, software updates during the very long study period, especially the introduction of iterative reconstruction in 2012, as well as inconsistent patient inspiration depth and scan length, make a reliable comparison of radiation parameters between groups impossible. Thirdly, we can only assume the nephroprotective effect of MBT due to the application of lesser amounts of a potentially nephrotoxic substance. Complete documentation of kidney function following CTA was not available. Therefore, it was not included in the final analysis. Last but not least, due to the retrospective study design, the possible influence of the location of the venous line and flow of CA application was not possible to analyze.

## Conclusion

All three analyzed chest CTA contrast injection protocols offered satisfying enhancement of the major thoracic vessels in patients younger than 24 months, while MBT demonstrated a reduction of image artifacts and CA amounts by a quarter in comparison to conventional CTA and fixed-bolus injection.

## Supplementary information

Table S1
