# Supplementary material for: Chest CTA in children younger than two years – a retrospective comparison of three contrast injection protocols
Source: Sci Rep. 2019 Dec 2;9:18109. doi: 10.1038/s41598-019-54498-z (PMC6889233; doi:10.1038/s41598-019-54498-z)
Supplement: Supplementary file 1 — Table S1 [file 41598_2019_54498_MOESM1_ESM.docx]

Chest CTA in children younger than two years – a retrospective comparison of three contrast injection protocols

Original research paper

Dr. Nagy Eszter^1*^, Dr. Tschauner Sebastian^1^, Dr. Marterer Robert^1^, Dipl.-Ing. Dr. Riedl Regina^2^, Prof. Dr. Sorantin Erich^1^

^1^ Division of Paediatric Radiology, Department of Radiology, Medical University Graz, Austria

^2^ Institute of Medical Informatics, Statistics and Documentation, Medical University Graz, Austria

## Supplementary material

## Table S1: Density measurements in different thoracic regions

| **Region** | **Variable** | **Group I**  **(n=32)**  **(mean ± SD)** | **Group II**  **(n=23)**  **(mean ± SD)** | **Group III**  **(n=15)**  **(mean ± SD)** | **p**  **(Group I vs II)** | **P**  **(Group I vs III)** | **P Group II vs III)** |
| --- | --- | --- | --- | --- | --- | --- | --- |
| **Ascending aorta** | *Enhancement* | 452.8 ± 221.2 | 599.7 ± 220.3 | 596.2 ± 221.3 | **0.001*** | **0.005^#^** | 0.927 |
|  | *Image noise* | 25.8 ± 13.7 | 36.7 ± 22.1 | 29.7 ± 14.4 | **0.010*** | 0.322 | 0.215 |
|  | *SNR* | 18.6 ± 6.9 | 21.2 ± 12.9 | 22.1 ± 12.4 | 0.347 | 0.263 | 0.768 |
|  | *CNR* | 30.1 ± 24.2 | 39.7 ± 29.2 | 39.1 ± 23.8 | 0.055 | 0.088 | 0.969 |
| **Aortic arch** | *Enhancement* | 455.1 ± 233.4 | 596.7 ± 202.4 | 581.8 ± 287.1 | **0.004*** | **0.014^#^** | 0.942 |
|  | *Image noise* | 28.8 ± 13.9 | 33.9 ± 20.5 | 28.2 ± 29.4 | 0.076 | 0.597 | 0.323 |
|  | *SNR* | 16.8 ± 7.9 | 21.3 ± 9.9 | 23.3 ± 12.7 | 0.118 | **0.042^#^** | 0.501 |
|  | *CNR* | 30.3 ± 24.8 | 39.5 ± 28.5 | 38.5 ± 24.2 | 0.073 | 0.121 | 0.988 |
| **Descending**  **aorta** | *Enhancement* | 448.0 ± 218.5 | 606.8 ± 236.4 | 565.1 ± 262.7 | **0.001*** | **0.017^#^** | 0.639 |
|  | *Image noise* | 27.4 ± 18.6 | 25.2 ± 11.7 | 25.6 ± 19.7 | 0.959 | 0.892 | 0.930 |
|  | *SNR* | 18.6 ± 8.7 | 26.6 ± 10.2 | 24.1 ± 12.0 | **0.005*** | 0.071 | 0.520 |
|  | *CNR* | 29.7 ± 23.4 | 40.5 ± 30.7 | 37.0 ± 22.0 | **0.041*** | 0.149 | 0.737 |
| **Superior cava vein** | *Enhancement* | 543.5 ± 369.5 | 766.1 ± 525.7 | 669.6 ± 433.6 | **0.033*** | 0.211 | 0.545 |
|  | *Image noise* | 126.9 ± 108.1 | 156.6 ± 200.0 | 129.2 ± 90.3 | 0.386 | 0.861 | 0.575 |
|  | *SNR* | 6.6 ± 4.4 | 8.6 ± 5.7 | 7.3 ± 6.6 | 0.229 | 0.784 | 0.453 |
|  | *CNR* | 38.5 ± 38.3 | 52.7 ± 41.2 | 44.5 ± 38.4 | 0.084 | 0.339 | 0.588 |
| **Pulmonary trunc** | *Enhancement* | 495.7 ± 294.9 | 580.6 ± 267.1 | 531.5 ± 303.2 | 0.070 | 0.248 | 0.674 |
|  | *Image noise* | 39.9 ± 43.0 | 43.2 ± 26.2 | 34.6 ± 19.4 | 0.477 | 0.942 | 0.504 |
|  | *SNR* | 15.0 ± 5.9 | 16.2 ± 8.7 | 15.5 ± 4.9 | 0.419 | 0.701 | 0.757 |
|  | *CNR* | 34.1 ± 31.8 | 38.5 ± 27.1 | 34.3 ± 25.4 | 0.219 | 0.469 | 0.734 |
| **Right atrium** | *Enhancement* | 467.1 ± 267.6 | 521.7 ± 238.6 | 442.5 ± 251.2 | 0.145 | 0.713 | 0.380 |
|  | *Image noise* | 63.7 ± 47.3 | 68.3 ± 51.9 | 56.9 ± 47.3 | 0.458 | 0.984 | 0.519 |
|  | *SNR* | 8.3 ± 2.0 | 9.4 ± 3.4 | 9.6 ± 3.6 | 0.213 | 0.196 | 0.837 |
|  | *CNR* | 31.9 ± 28.7 | 34.1 ± 23.1 | 28.6 ± 22.7 | 0.337 | 0.798 | 0.572 |
| **Right ventricle** | *Enhancement* | 426.7 ± 235.7 | 520.3 ± 227.1 | 467.9 ± 285.1 | **0.041*** | 0.225 | 0.578 |
|  | *Image noise* | 51.9 ± 37.7 | 63.3 ± 53.5 | 42.0 ± 34.2 | 0.131 | 0.864 | 0.155 |
|  | *SNR* | 9.5 ± 3.0 | 10.3 ± 4.0 | 13.1 ± 5.1 | 0.699 | **0.013^#^** | **0.036°** |
|  | *CNR* | 28.4 ± 25.3 | 34.0 ± 23.2 | 30.7 ± 26.3 | 0.159 | 0.353 | 0.772 |
| **Left atrium** | *Enhancement* | 431.2 ± 229.5 | 578.4 ± 195.9 | 573.2 ± 290.8 | **0.001*** | **0.005^#^** | 0.939 |
|  | *Image noise* | 31.9 ± 13.8 | 48.8 ± 25.2 | 48.4 ± 26.5 | **0.002*** | **0.005^#^** | 0.971 |
|  | *SNR* | 13.5 ± 4.0 | 13.6 ± 5.2 | 12.2 ± 3.7 | 0.698 | 0.494 | 0.328 |
|  | *CNR* | 28.6 ± 26.9 | 38.2 ± 26.2 | 37.6 ± 24.2 | 0.056 | 0.089 | 0.972 |
| **Left ventricle** | *Enhancement* | 423.6 ± 211.3 | 572.8 ± 213.4 | 541.2 ± 251.7 | **0.001*** | **0.009^#^** | 0.748 |
|  | *Image noise* | 40.3 ± 22.0 | 51.9 ± 24.8 | 45.9 ± 28.8 | **0.038*** | 0.286 | 0.467 |
|  | *SNR* | 11.2 ± 4.5 | 13.5 ± 8.9 | 12.3 ± 3.6 | 0.099 | 0.309 | 0.682 |
|  | *CNR* | 28.1 ± 24.4 | 37.7 ± 28.4 | 35.3 ± 21.0 | 0.052 | 0.132 | 0.850 |
| **Right upper pulmonary vein** | *Enhancement* | 420.4 ± 224.5 | 523.8 ± 169.6 | 537.4 ± 273.9 | 0.051 | 0.486 | 0.663 |
|  | *Image noise* | 24.4± 11.6 | 30.7 ± 19.0 | 27.2 ± 14.3 | **0.036*** | 0.204 | 0.627 |
|  | *SNR* | 19.4 ± 9.4 | 21.7 ± 12.5 | 22.1 ± 9.6 | 0.493 | 0.431 | 0.835 |
|  | *CNR* | 27.6 ± 25.6 | 34.0 ± 17.8 | 33.9 ± 21.8 | 0.171 | 0.178 | 0.846 |
| **Right lower pulmonary vein** | *Enhancement* | 414.9 ± 205.0 | 522.7 ± 149.0 | 530.4 ± 271.3 | 0.092 | 0.606 | 0.884 |
|  | *Image noise* | 22.0 ± 7.6 | 25.0 ± 12.0 | 22.8 ± 10.1 | 0.101 | 0.344 | 0.654 |
|  | *SNR* | 19.3 ± 7.7 | 24.3 ± 10.1 | 24.6 ± 12.5 | 0.052 | 0.101 | 0.981 |
|  | *CNR* | 27.5 ± 25.4 | 33.3 ± 16.2 | 33.9 ± 21.0 | 0.148 | 0.178 | 0.912 |
| **Left upper pulmonary vein** | *Enhancement* | 406.0 ± 193.0 | 549.8 ± 210.0 | 542.1 ± 285.3 | 0.110 | 0.766 | 0.983 |
|  | *Image noise* | 25.0 ± 18.0 | 27.5 ± 8.4 | 32.4 ± 18.4 | 0.341 | 0.091 | 0.385 |
|  | *SNR* | 18.6 ± 8.0 | 21.3 ± 10.6 | 17.0 ± 4.2 | 0.214 | 0.728 | 0.172 |
|  | *CNR* | 26.0 ± 22.3 | 34.5 ± 15.9 | 34.9 ± 22.7 | 0.063 | 0.083 | 0.886 |

| **Left lower pulmonary vein** | *Enhancement* | 408.4 ± 234.0 | 543.2 ± 200.2 | 532.5 ± 280.5 | 0.106 | 0.719 | 0.994 |
| --- | --- | --- | --- | --- | --- | --- | --- |
|  | *Image noise* | 23.7 ± 11.9 | 26.6 ± 9.6 | 26.9 ± 15.3 | 0.148 | 0.123 | 0.784 |
|  | *SNR* | 18.2 ± 7.6 | 22.0 ± 8.7 | 20.8 ± 6.3 | 0.092 | 0.308 | 0.655 |
|  | *CNR* | 27.3 ± 28.9 | 33.6 ± 14.8 | 34.1 ± 21.7 | 0.207 | 0.199 | 0.859 |
| **Liver** | *Enhancement* | 89.2 ± 21.7 | 84.2 ± 16.7 | 93.7 ± 27.3 | 0.688 | 0.283 | 0.175 |
|  | *Image noise* | 18.3 ± 4.2 | 17.7 ± 6.2 | 19.7 ± 5.2 | 0.906 | 0.294 | 0.272 |
|  | *SNR* | 5.0 ± 1.2 | 5.1 ± 1.4 | 5.0 ± 1.7 | 0.675 | 0.931 | 0.787 |
|  | *CNR* | 2.0 ± 1.5 | 1.8 ± 1.4 | 2.4 ± 2.4 | 0.867 | 0.356 | 0.308 |
| **Muscle** | *Enhancement* | 61.3 ± 10.4 | 58.5 ± 11.1 | 62.1 ± 9.1 | 0.560 | 0.548 | 0.289 |
|  | *Image noise* | 14.2 ± 3.4 | 16.7 ± 10.5 | 14.4 ± 3.4 | 0.115 | 0.721 | 0.327 |
|  | *SNR* | 4.6 ± 1.7 | 4.1 ± 1.6 | 4.6 ± 1.6 | 0.424 | 0.791 | 0.358 |
|  | *CNR* | - | - | - | - | - |  |

1. ***** significant difference between group I and II, # significant difference between group I and III, § significant difference between group II and III
